# Eocene amber provides the first fossil record and bridges distributional gap in the rare genus *Robsonomyia* (Diptera: Keroplatidae)

**DOI:** 10.1038/s41598-024-59448-y

**Published:** 2024-04-22

**Authors:** Alicja Pełczyńska, Wiesław Krzemiński, Vladimir Blagoderov, Lars Vilhelmsen, Agnieszka Soszyńska

**Affiliations:** 1 Faculty of Biology and Environmental Protection, Department of Invertebrate Zoology and Hydrobiology, University of Lodz, 90-237 Lodz, Poland; 2 Institute of Systematics and Evolution of Animals, Polish Academy of Sciences, 31-016 Kraków, Poland; 3 National Museums Scotland, Edinburgh, Scotland; 4 Natural History Museum of Denmark, SCIENCE, University of Copenhagen, Universitetsparken 15, 2100 Copenhagen, Denmark

**Keywords:** Zoology, Entomology, Evolution, Palaeontology, Taxonomy

## Abstract

Until now, the genus *Robsonomyia* was represented by two extant species: *R. reducta* Matile & Vockeroth, 1980 from North America and *R. sciaraeformis* (Okada, 1939) from Asia. This paper presents the first fossil members of the genus *Robsonomyia*, which is also the first record from Europe. Two new fossil species from Baltic amber are described: *R. baltica* Pełczyńska, Krzemiński & Blagoderov, sp. nov. and *R. henningseni* Pełczyńska, Krzemiński & Blagoderov, sp. nov.. The presence of fossil *Robsonomyia* spp. on the European continent suggests Holarctic distribution of the genus in the past. We also discuss possible pathways of its intercontinental dispersion.

## Introduction

The family Keroplatidae Rondani, 1856, commonly known as “predatory fungus gnats”, is one of the largest and most diverse families of the dipteran infraorder Bibionomorpha. Keroplatidae has a worldwide distribution and comprises nearly 1000 extant species in almost 100 different genera^1,2^. The biology and ecology of the members of this family vary greatly^3^. In most genera, the larvae are carnivorous and use a sticky web covered with acidic fluid to capture and kill small invertebrates, e.g. imagines of other Diptera. In addition to this, some of them use bioluminescence as a lure for phototropic insect prey. Mycophagy and sporophagy also occur. In some genera, scavenging, cannibalism, and even endoparasitism have been observed. The diet of adults in most genera remains unknown, although feeding on flower nectar has been observed in some^1,2,4^.

Keroplatidae appeared in the fossil record as early as the Lower Cretaceous. The oldest keroplatid was found in sedimentary rocks of the Middle Purbeck at Durlston Bay (England) and dates back to the Berriasian (~ 140 Ma). Unfortunately, this specimen remains undescribed due to the poor state of preservation preventing the observation of crucial diagnostic characters^5,6^. The oldest described species *Lebanognoriste prima* Blagoderov & Grimaldi, 2004 was found in Lebanese amber dated the late Barremian (~ 125 Ma)^7^. Additional keroplatid species from the Lower Cretaceous were identified in Burmese amber from Myanmar (comprising 12 species) and Escucha amber from Spain (comprising two species) (Table 1). To date, a total of 71 fossil species have been described (Table 1), including Adamacrocerinae (one species), Keroplatinae (17 species), Lygistorrhininae (16 species) Macrocerinae (18 species), Platyurinae (two species), and one species from a genus not yet assigned to a specific subfamily (*Vladelektra blagoderovi* Evenhuis, 2020). In addition, there are 16 species with uncertain taxonomic position and new species are continuously being described, which indicates that our understanding of the diversity within this family is still incomplete and further revision is needed^8^. The majority of Keroplatidae fossils are found in Baltic amber from the Eocene^9^. Until now, 35 species from nine different genera have been documented in this fossil resin (Table 1).

**Table 1:** Known fossil species of the Keroplatidae.

No.	Species name	Time	Locality	Type
1	*Adamacrocera adami* Ševčík et al. 2020	Cretaceous (Albian/Cenomanian)	OR: Burmese amber (Myanmar)	A
2	*Archaeognoriste primitiva* Blagoderov & Grimaldi, 2004	Cretaceous (Albian/Cenomanian)	OR: Burmese amber (Myanmar)	A
3	*Asindulum elegantulum* Meunier, 1904	Middle/Late Eocene (Lutetian/Priabonian)	PA: Baltic amber	A
4	*Asindulum girschneri* Meunier, 1904	Middle/Late Eocene (Lutetian/Priabonian)	PA: Baltic amber	A
5	*Asindulum longipalpe* Meunier, 1904	Middle/Late Eocene (Lutetian/Priabonian)	PA: Baltic amber	A
6	*Asindulum pygmaeum* Statz, 1944	Oligocene (Chattian)	PA: Rott Formation (Germany)	C
7	*Burmacrocera petiolata* Cockerell, 1917	Cretaceous (Albian/Cenomanian)	OR: Burmese amber (Myanmar)	A
8	*Eomacroceritis melanopoda* (Hong 1974)	Eocene (Ypresian)	OR: Fushun Amber (China)	A
9	*Eoplatyura noda* (Hong, 1981)	Eocene (Ypresian)	OR: Fushun Amber (China)	A
10	*Hegalari antzinako* Blagoderov & Arillo, 2002	Cretaceous (Albian)	PA: Escucha amber (Spain)	A
11	*Hegalari minor* Blagoderov & Arillo, 2002	Cretaceous (Albian)	PA: Escucha amber (Spain)	A
12	*Hesperodes concinna* (Meunier, 1917)	Middle/Late Eocene (Lutetian/Priabonian)	PA: Baltic amber	A
13	*Indorrhina sahnii* Stebner & Grimaldi, 2017	Eocene (Ypresian)	OR : Cambay amber (India)	A
14	*Kelneria abundare* (Meunier, 1904)	Middle/Late Eocene (Lutetian/Priabonian)	PA: Baltic amber	A
15	*Kelneria ciliata* (Meunier, 1904)	Middle/Late Eocene (Lutetian/Priabonian)	PA: Baltic amber	A
16	*Kelneria filiformis* (Meunier, 1904)	Middle/Late Eocene (Lutetian/Priabonian)	PA: Baltic amber	A
17	*Kelneria setosa* Matile, 1979	Middle/Late Eocene (Lutetian/Priabonian)	PA: Baltic amber	A
18	*Lebanognoriste prima* Blagoderov & Grimaldi, 2004	Cretaceous (Late/Upper Barremian)	PA: Lebanon amber	A
19	*Leptognoriste davisi* Blagoderov & Grimaldi, 2004	Cretaceous (Albian/Cenomanian)	OR: Burmese amber (Myanmar)	A
20	*Leptognoriste microstoma* Blagoderov & Grimaldi, 2004	Cretaceous (Albian/Cenomanian)	OR: Burmese amber (Myanmar)	A
21	*Lygistorrhina caribbiana* Grund, 2012	Miocene (Burdigalian/Langhian)	NT: Dominican amber (Dominican Republic)	A
22	*Lygistorrhina indica* Stebner and Grimaldi, 2017	Eocene (Ypresian)	OR: Cambay amber (India)	A
23	*Macrocera apithanos* Kerr and Greenwalt, 2022	Eocene (Lutetian)	NE: Kishenehn Formation of Montana (USA)	C
24	*Macrocera archaica* (Armbruster, 1938)	Miocene (Langhian)	PA: Randeck Maar Formation (Germany)	C
25	*Macrocera electricornis* Evenhuis, 2006	Middle/Late Eocene (Lutetian/Priabonian)	PA: Baltic amber	A
26	*Macrocera soccata* Meunier, 1899	Middle/Late Eocene (Lutetian/Priabonian)	PA: Baltic amber	A
27	*Macrocera umbonata* Statz, 1944	Oligocene (Chattian)	PA: Rott Formation (Germany)	C
28	*Micrepimera neli* Blagoderov & Skibińska 2019	Middle/Late Eocene (Lutetian/Priabonian)	PA: Baltic amber	A
29	*Micrepimera elegantissima* (Meunier 1904)	Middle/Late Eocene (Lutetian/Priabonian)	PA: Baltic amber	A
30	*Palaeoasindulum curvipalpe* (Meunier, 1904)	Middle/Late Eocene (Lutetian/Priabonian)	PA: Baltic amber	A
31	*Palaeognoriste affine* Meunier, 1912	Middle/Late Eocene (Lutetian/Priabonian)	PA: Baltic amber	A
32	*Palaeognoriste orientale* Stebner & Grimaldi, 2017	Eocene (Ypresian)	OR: Cambay amber (India)	A
33	*Palaeognoriste sciariforme* Meunier 1904	Middle/Late Eocene (Lutetian/Priabonian)	PA: Baltic amber	A
34	*Paleoplatyura agnieszkae* Ševčík, Krzemiński & Skibińska 2021	Cretaceous (Albian/Cenomanian)	OR: Burmese amber (Myanmar)	A
35	*Paleoplatyura eocenica* Cockerell, 1921	Eocene (Ypresian)	NE: Green River Formation (USA)	C
36	*Paleoplatyura loewi* (Meunier, 1922)	Middle/Late Eocene (Lutetian/Priabonian)	PA: Baltic amber	A
37	*Paleoplatyura macrocera* (Meunier, 1899)	Middle/Late Eocene (Lutetian/Priabonian)	PA: Baltic amber	A
38	*Paleoplatyura magnifica* Ševčík, Krzemiński & Skibińska 2021	Cretaceous (Albian/Cenomanian)	OR: Burmese amber (Myanmar)	A
39	*Paleoplatyura miae* Ševčík, Krzemiński & Skibińska 2021	Cretaceous (Albian/Cenomanian)	OR: Burmese amber (Myanmar)	A
40	*Parisognoriste eocenica* Blagoderov, Hippa & Nel, 2010	Lowermost Eocene	PA : Oise Amber (France)	A
41	*Platyura calcar* Meunier, 1899	Middle/Late Eocene (Lutetian/Priabonian)	PA: Baltic amber	A
42	*Platyura conjuncta* Loew, 1850	Middle/Late Eocene (Lutetian/Priabonian)	PA: Baltic amber	A
43	*Plesiognoriste carpenteri* Blagoderov & Grimaldi, 2004	Cretaceous (Santonian)	NE: Cedar Lake amber (Canada)	A
44	*Plesiognoriste zherikhini* Blagoderov & Grimaldi, 2004	Creataceus (Coniacian/Santonian)	PA: Taimyr amber, Yantardakh (Russia)	A
45	*Proapemon infernus* Melander, 1949	Eocene (Prabonian)	NE: Florissant Formation (USA)	C
46	*Proceroplatus hennigi* Schmalfuss, 1979	Miocene (Burdigalian/Langhian)	Nt: Dominican amber (Dominican Republic)	A
47	*Proceroplatus preziosii* Evenhuis & Penney, 2013	Miocene (Burdigalian/Langhian)	NT: Dominican amber (Dominican Republic)	A
48	*Protognoriste amplicauda* Blagoderov & Grimaldi, 2004	Cretaceous (Albian/Cenomanian)	PA: Taimyr amber Nizhnaya Agapa (Russia)	A
49	*Protognoriste goeleti* Blagoderov & Grimaldi, 2004	Cretaceous (Albian/Cenomanian)	OR: Burmese amber (Myanmar)	A
50	*Protognoriste nascifoa* Blagoderov & Grimaldi, 2004	Cretaceous (Albian/Cenomanian)	OR: Burmese amber (Myanmar)	A
51	*Robsonomyia baltica* Pełczyńska, Krzemiński et Blagoderov, sp. nov	Middle/Late Eocene (Lutetian/Priabonian)	PA: Baltic amber	A
52	*Robsonomyia henningseni* Pełczyńska, Krzemiński et Blagoderov, sp. nov	Middle/Late Eocene (Lutetian/Priabonian)	PA: Baltic amber	A
53	*Schlueterimyia cenomanica* Matile, 1981	Cretaceous	PA: Bezonnais (Ecommoy) amber (France)	A
54	*Vastaplatyura electrica* Solórzano-Kraemer & Evenhuis 2008	Eocene (Ypresian)	OR: Cambay amber (India)	A
55	*Vladelektra blagoderovi* Evenhuis, 2020	Eocene (Ypresian)	OR: Burmese amber (Myanmar)	A
Keroplatidae incertae sedis				
56	*?Platyura armata* Meunier, 1899	Middle/Late Eocene (Lutetian/Priabonian)	PA: Baltic amber	A
57	*?Platyura ceroplatites* Meunier, 1904	Middle/Late Eocene (Lutetian/Priabonian)	PA: Baltic amber	A
58	*?Platyura ceroplatoides* Meunier, 1904	Middle/Late Eocene (Lutetian/Priabonian)	PA: Baltic amber	A
59	*?Platyura crassicornis* Meunier, 1917	Middle/Late Eocene (Lutetian/Priabonian)	PA: Baltic amber	A
60	*?Platyura distincta* Meunier, 1904	Middle/Late Eocene (Lutetian/Priabonian)	PA: Baltic amber	A
61	*?Platyura ectorsii* Meunier, 1904	Middle/Late Eocene (Lutetian/Priabonian)	PA: Baltic amber	A
62	*?Platyura ehrhardti* Loew, 1850	Middle/Late Eocene (Lutetian/Priabonian)	PA: Baltic amber	A
63	*?Platyura exigua* Meunier, 1907	Pleistocene/Holocene	PA: Tanzania	K
64	*?Platyura graciosa* Meunier, 1904	Middle/Late Eocene (Lutetian/Priabonian)	PA: Baltic amber	A
65	*?Platyura kunowi* Meunier, 1904	Middle/Late Eocene (Lutetian/Priabonian)	PA: Baltic amber	A
66	*?Keroplatus major* Meunier, 1904	Middle/Late Eocene (Lutetian/Priabonian)	PA: Baltic amber	A
67	*?Platyura mikii* Meunier, 1904	Middle/Late Eocene (Lutetian/Priabonian)	PA: Baltic amber	A
68	*?Platyura moniliforfis* Meunier, 1904	Middle/Late Eocene (Lutetian/Priabonian)	PA: Baltic amber	A
69	*?Platyura obliqua* (Cockerell 1921)	Eocene (Priabonian)	PA: Bouldnor Formation (UK)	C
70	*?Platyura pusilla* Meunier, 1899	Middle/Late Eocene (Lutetian/Priabonian)	PA: Baltic amber	A
71	*?Platyura verrali* Meunier, 1904	Middle/Late Eocene (Lutetian/Priabonian)	PA: Baltic amber	A

NT, Neotropical; OR, Oriental; PA, Palaearctic; NE, Nearctic; A, Amber fossil; C, Compression fossil; K, Copal.

The genus *Robsonomyia* Matile, 1980 belongs to the Macrocerinae. This subfamily comprises two tribes: Robsonomyiini and Macrocerini^10^. Previous phylogenetic analyses, primarily based on morphological characters were conducted by Matile (1990) for Keroplatidae and by Ševčík (2009) for the Robsonomyiini^10,11^. These analyses suggested the monophyly of this subfamily. However, a more comprehensive recent molecular analysis of Keroplatidae by Mantic et al*.* (2020), was performed using maximum likelihood (ML) and Bayesian methods (BI); the ML analyses tentatively supported the monophyly of the Macrocerinae, albeit with limited statistical support, while the BI analyses indicated that they are paraphyletic^2^.

The Robsonomyinii encompasses six genera, five of which have modern representatives: *Calusamyia* Coher, 2011; *Robsonomyia* Matile, 1980; *Micrepimera* Matile, 1990; *Srilankana* Matile, 1990; *Langkawiana* Ševčík, 2009. Additionally, *Kelneria* Matile, 1979 is known only from fossils^11,12^. The biology of Robsonomyinii remains largely unexplored. The developmental habitat of their larvae and the characteristics of the females are unknown, which is a common state in many genera within the Keroplatidae^2,10,13^. A distinctive feature of Robsonomyiini is the reduction in radial wing venation, and a unique apomorphy: a membranous area that separates the ocellar sclerite from the frons. Furthermore, they have a reduced vertical mesepimeron in the thoracic pleura^10,12^.

*Robsonomyia* is distinguished from other members of the tribe by the shape of Sc vein which ends on Rb, instead of terminating in the costa^14^. *Robsonomyia* is currently represented by only two extant species, with a geographically disjunct distribution. *R. reducta* Matile & Vockeroth, 1980 is found in western North America, specifically in California (USA), and British Columbia (Canada)^14^. The other species, *R. sciaraeformis* (Okada, 1939), is native to East Asia, with occurrences in Japan, particularly in Sapporo, Hokkaido Island^15^. In this paper, we aim to shed light on the distributional history of *Robsonomyia* by incorporating new insights obtained from the discovery of their fossil representatives preserved in the Baltic amber.

## Results

### Systematic Palaeontology

- **Order** Diptera Linnaeus, 1758
- **Infraorder** Bibionomorpha Hennig, 1948
- **Superfamily** Sciaroidea Billberg, 1820
- **Family** Keroplatidae Rondani, 1856
- **Subfamily** Macrocerinae Rondani, 1856
- **Tribe** Robsonomyiini Matile, 1990
- **Genus** *Robsonomyia* Matile & Vockeroth, 1980

Type species *Robsonomyia reducta* Matile & Vockeroth, 1980.

The genus includes two extant species: *Robsonomyia reducta* Matile & Vockeroth, 1980, *Robsonomyia sciaraeformis* (Okada, 1939) and two fossil: *Robsonomyia baltica* Pełczyńska, Krzemiński et Blagoderov, sp. nov., and *Robsonomyia henningseni* Pełczyńska, Krzemiński et Blagoderov, sp. nov.

**Figure 1:**
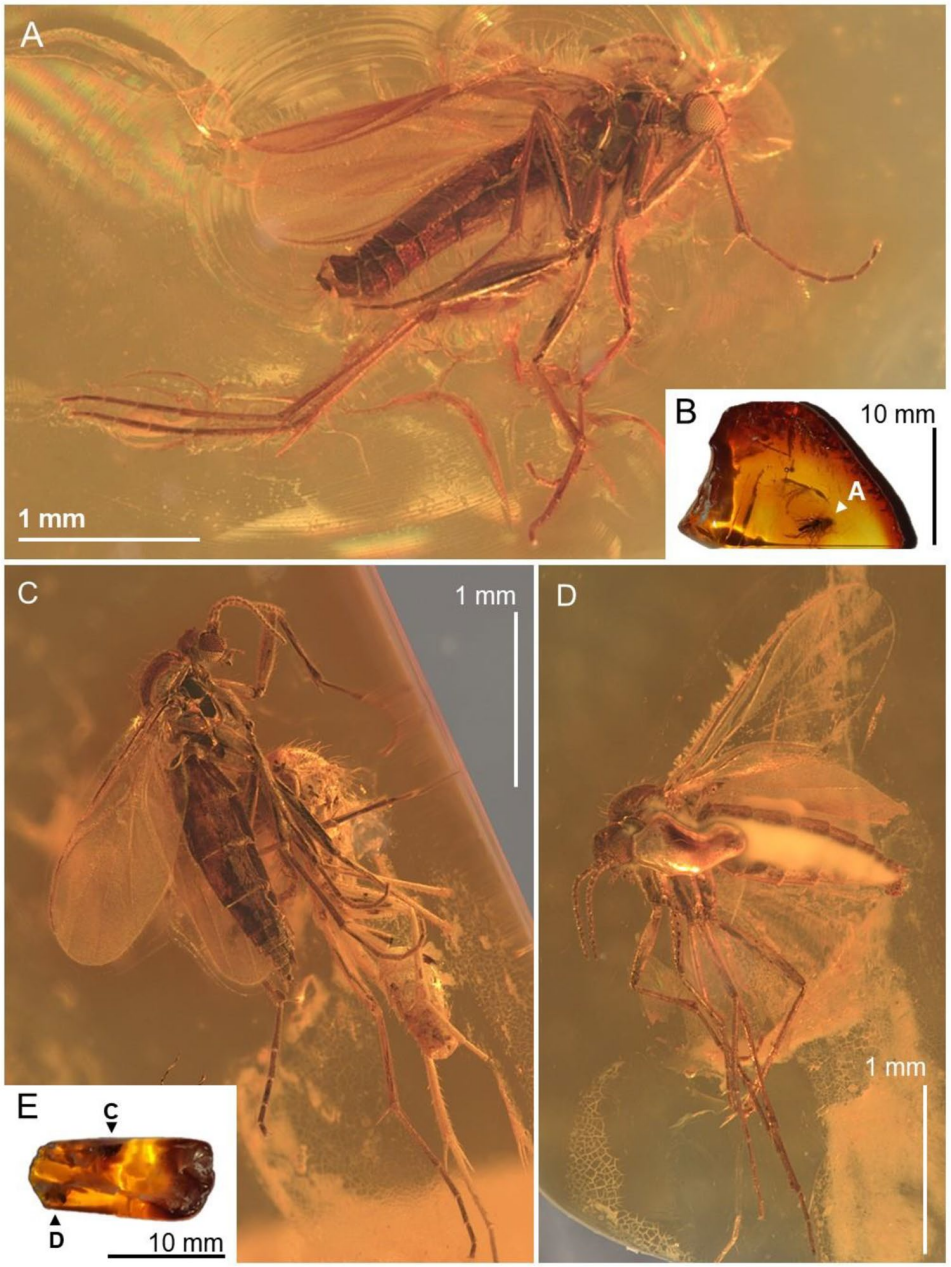
*Robsonomyia baltica* sp. nov. (NHMD-300551): (**A**) male (holotype No NHMD-300551a); (**B**) amber piece with position of male; (**C**) female (paratype No NHMD-300551b); (**D**) female (paratype No NHMD-300551c); (**E**) amber piece with position of females.

**Figure 2:**
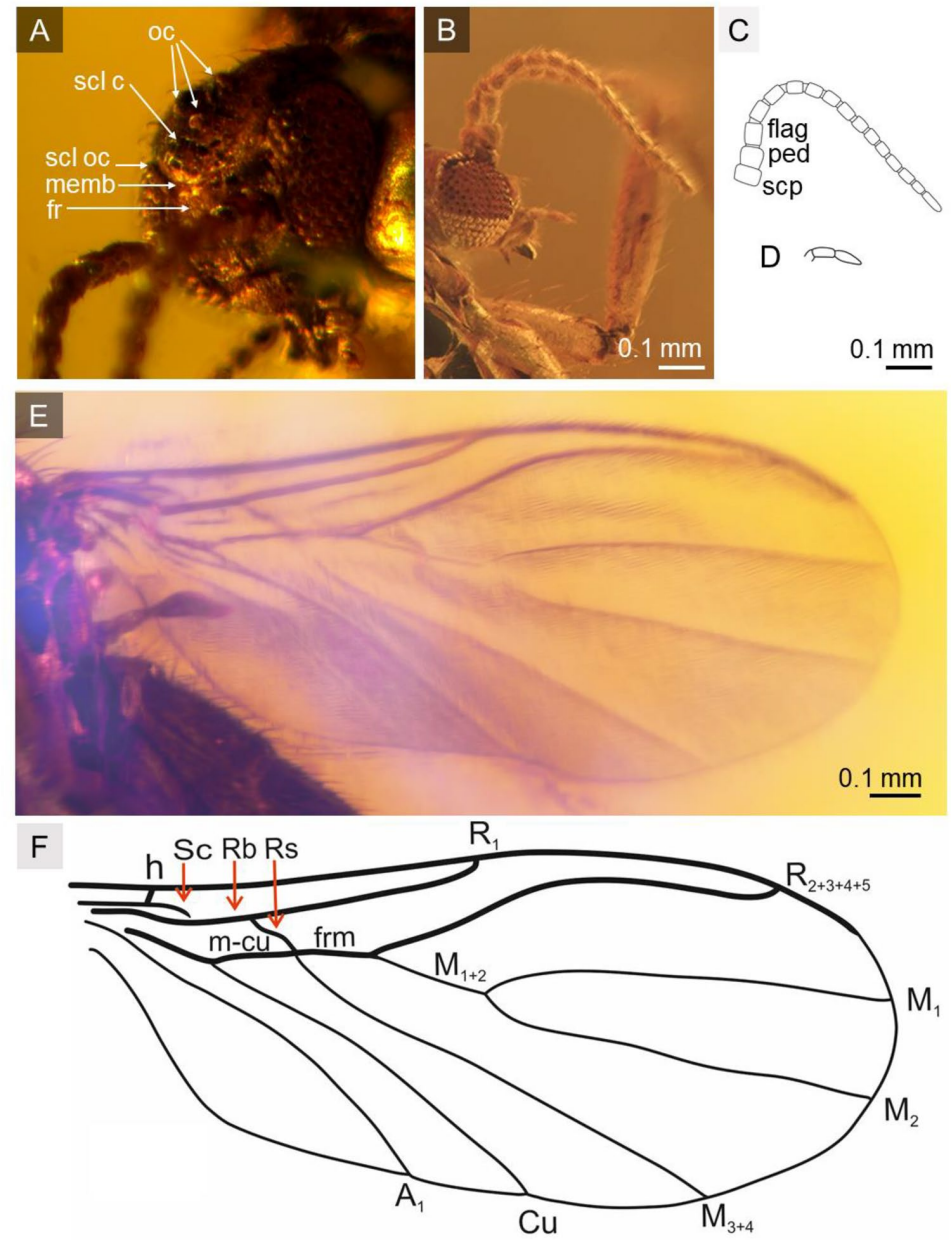
*Robsonomyia baltica* sp. nov. (NHMD-300551c): (**A**) head (abbreviations: oc = ocelli; scl c = cerebral sclerite; scl oc = ocellar sclerite; memb = membranous area; fr = frons). *Robsonomyia baltica* sp. nov. (NHMD-300551b): (**B**) head in lateral view; (**C**) antennae (abbreviations: scp = scapus, ped = pedicel, flag = flagellum); (**D**) palpi; (**E**) wing; (F) wing venation (abbreviations: Sc = subcostal vein; h = humeral cross-vein; Rb = radio-basal vein; Rs = radial sector; R_1_ = anterior branch of radius; R_2+3+4+5_ = third branch of radius; *frm* = radio-medial fusion; *m-cu* = medial cubital crossvein; M_1+2_ = stem of media; M_1_ = first branch of media; M_2_ = second branch of media; M_3+4_ = fourth branch of media; Cu = cubital vein; A_1_ = first branch of anal vein).

**Figure 3:**
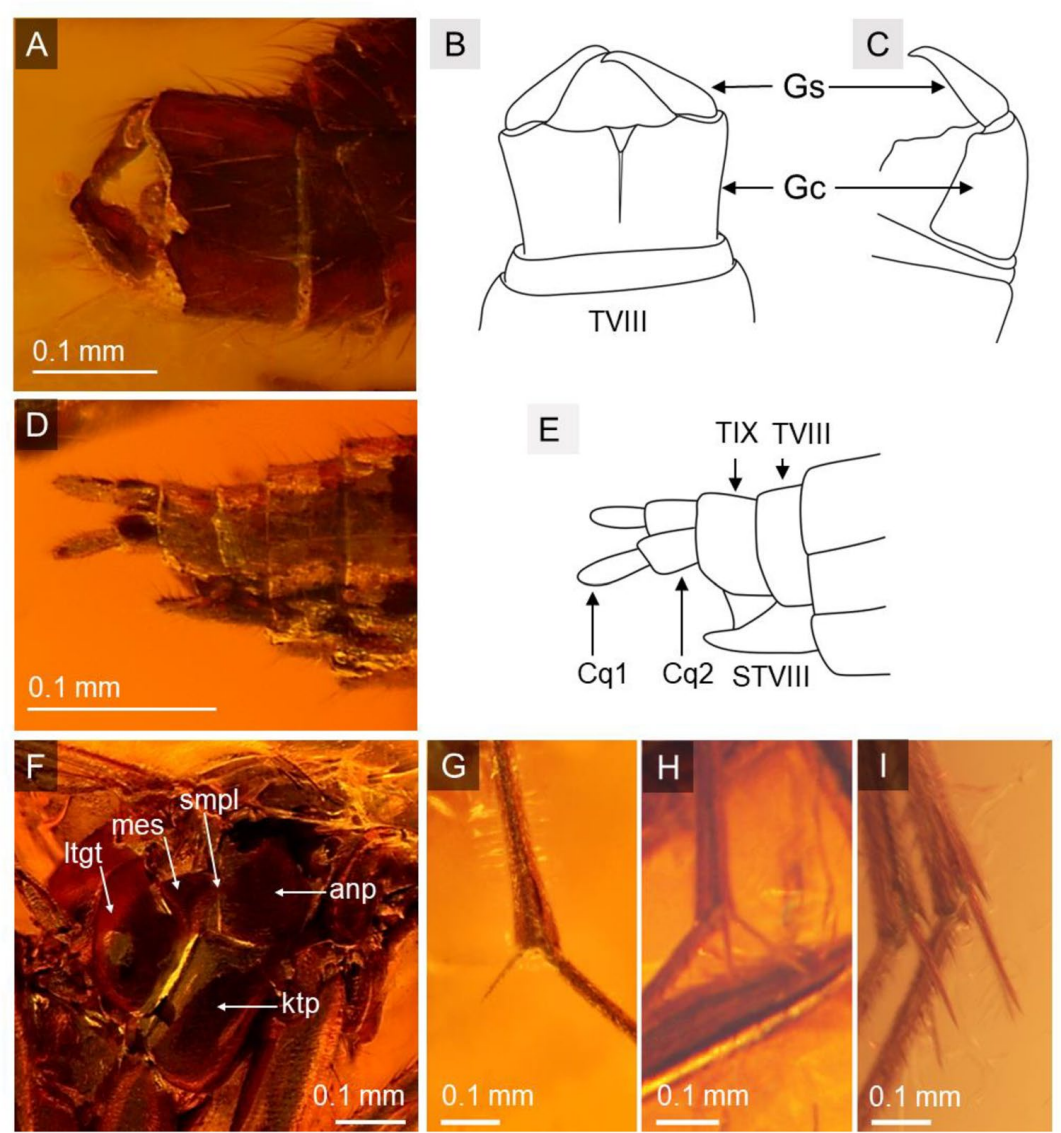
*Robsonomyia baltica* sp. nov. (NHMD-300551a): (**A**, **B**) male genitalia from dorsal side (abbreviations: Gs = gonostylusy; Gc = gonocoxites; TVIII = tergite VIII); (**C**) male genitalia from ventral side (abbreviations: Gs = gonostylusy; Gc = gonocoxite); (**G**) apical spur on fore tibia; (**H**) spurs on mid tibia; (I) spurs on hind tibia. *Robsonomyia baltica* sp. nov. (NHMD-300551b): (**D**, **E**) female genitalia. *Robsonomyia baltica* sp. nov. (NHMD-300551a): (**F**) thorax (abbreviations: anp = anepisternum; ktp = katepisternum; smpl = mediopleural suture; mes = mesepimeron; ltgt = laterotergite).

***Robsonomyia baltica*** Pełczyńska, Krzemiński et Blagoderov, **sp. nov.** (Figs. 1, 2, 3).

urn:lsid:zoobank.org:act:9B8A52F8-ED8B-41EA-86EB-0658D8C06131.

**Etymology:** The specific name refers to the Baltic region, where the fossil resin containing this species (Baltic amber) was found.

**Type material:** Baltic amber inclusion No NHMD-300551a (Fig. 1A); holotype (male) preserved in 19 × 13 × 4 mm piece of amber (Fig. 1B); paratypes No NHMD-300551b, No NHMD-300551c (two females) (Fig. 1C, D) preserved in 17 × 6 × 5 mm piece of amber (Fig. 1E).

**Diagnosis:** Sc very short, weakens considerably just before ending in Rb vein; vein R_1_ terminates distinctly before M_1+2_ forking into M_1_ and M_2_; vein M_1_ 3.5 × longer than M_1+2_; vein R_2+3+4+5_ strongly arched from half of its length towards anterior of the wing; vein M_3+4_ joins with m-cu almost opposite Rs; gonostylus as long as gonocoxites, conical, curved mesally just before the end, pointed at apex.

**Description:** Male NHMD-300551a (Fig. 1A): body 2.2 mm long, wing length 1.7 mm, antennae 0.8 mm long. Female NHMD-300551b (Fig. 1C): body 2.1 mm long, wing length 1.7 mm, antennae 0.7 mm long. Female NHMD-300551c (Fig. 1D): body 1.9 mm long, wing length 1.9 mm, antennae 0.6 mm long. Head (Fig. 2A, B): subspherical, wider than long; eyes large, well separated; membranous area separates ocellar sclerite and frons; large distinct cerebral sclerite present; three ocelli forming equilateral triangle. Antennae (Fig. 2B, C): relatively short, half of wing length, about 0.4 × as long as body; scapus slightly shorter than broad, 1.8 × wider than flagellomeres; pedicel with bulbous apical part, narrower than scape, 1.4 × wider than flagellomeres; flagellum 14 segmented; flagellomeres cylindrical, densely covered with short setae, almost as long as broad; terminal flagellomere slightly elongate, evenly tapered to rounded apex, 1.8 × longer than proximal flagellomeres. Palpi (Fig. 2D): with three visible maxillary palpomeres approx. as long as broad, last two segments of same length. Wings (Fig. 2E, F): 2.3 × longer than wide, membrane hyaline without microtrichiae and without any visible markings; costa ending halfway between R_2+3+4+5_ and M_1_; Sc very short and strongly curved towards radio basal vein (Rb), basal part thick and distinct, gradually becoming thinner, reaching Rb, before level of m-cu reaching Cu; R_1_ ending on mid-length of anterior margin of wing, nearly before level of M_1+2_ forking into M_1_ and M_2_; R_2+3+4+5_ strongly arched anteriorly, second half of the vein runs in parallel with C; m-cu vein approx. same length as radio-medial fusion (frm), veins continuous straight line; Rs distinct, oblique, nearly in one line with M_1+2_; Mb absent; M_1+2_ 1,7 × longer than frm, ending approx. at level of terminations of Cu; M_1_ 3.4 times as long as M_1+2_; Cu and A_1_ reaching wing margin; A_2_ absent. Thorax (Fig. 3F): about as high as long, scutum densely covered with long and thick hairs; anepisternum and katepisternum bare; mediopleural suture almost straight and subvertical; mesepimeron and laterotergite bare; mediotergite round and bare; haltere longer than first abdominal segment. Legs (Fig. 3G-I): fore coxa densely covered with long hair-like setae, mid coxa with sparse setae, hind coxa without visible setae; femora densely covered with short, robust setae; fore tibia with single apical spur, anterior tibial comb absent, mid and hind tibia with two equal length spurs, more robust and longer on hind tibia. Abdomen: densely covered with long hairs, all eight segments visible, I segment very short, segments II-IV approx. same length, following segments gradually decreasing in length, segment VIII retracted into VII; male terminalia (Fig. 3A-C): gonocoxites massive, fused ventrally, almost straight at the apical margin ventrally; gonostyli cylindrical, slightly curved, pointed at apex; aedeagus and the associated internal structures not visible; female terminalia (Fig. 3D, E): cercus two-segmented; basal segment tubular, 1,3 × longer than wide; apical segment elongated, oval, 2,7 × longer than wide.

**Figure 4:**
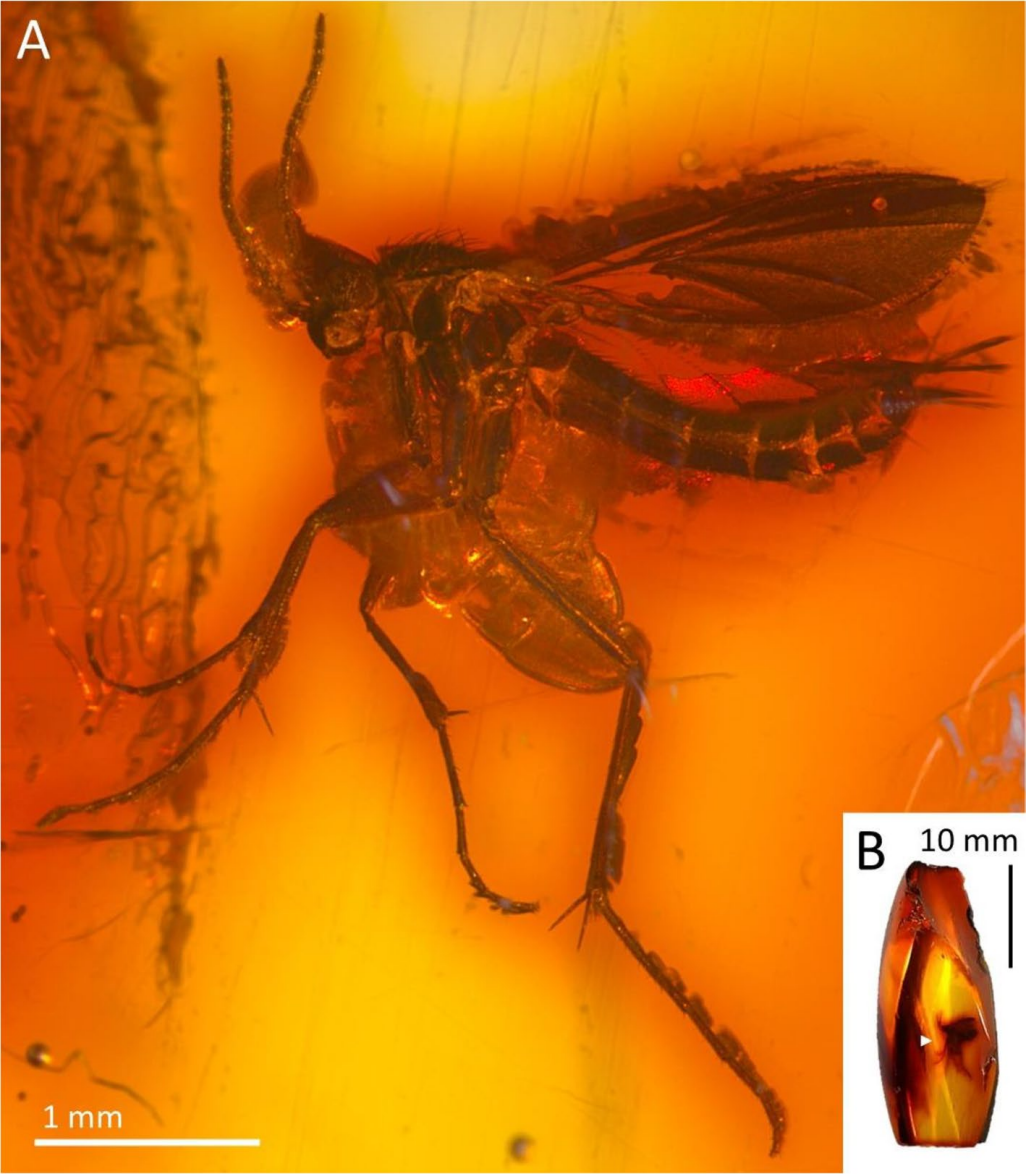
*Robsonomyia henningseni* sp. nov. (NHMD-39356): (**A**) male (holotype No NHMD-39356); (**B**) amber piece with position of male.

**Figure 5:**
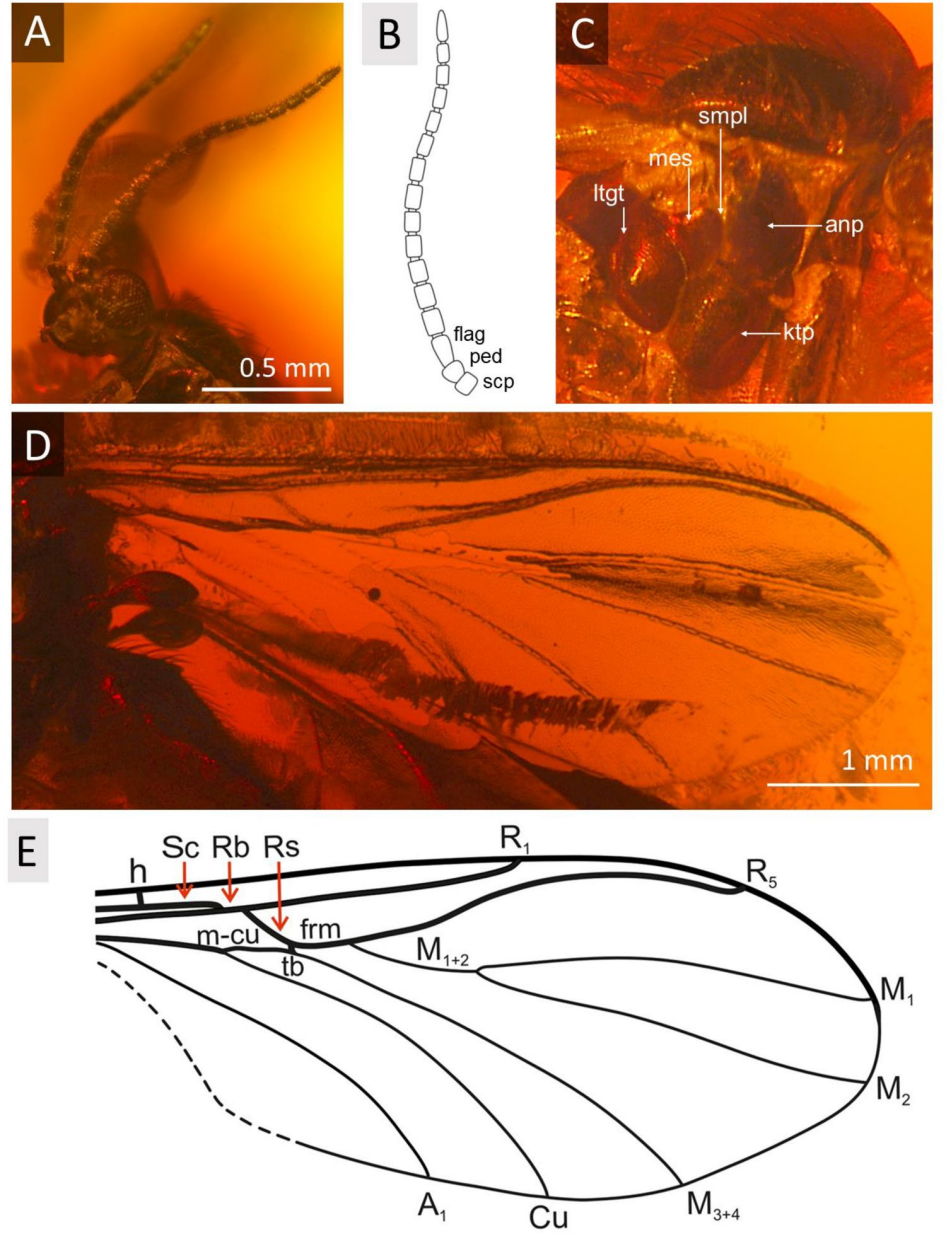
*Robsonomyia henningseni* sp. nov. (NHMD-39356): (**A**) head; (**B**) antennae; (**C**) thorax in lateral view; (**D**) wing; (**E**) wing venation (abbreviations: tb = transverse basale = basal part of M_3+4_).

**Figure 6:**
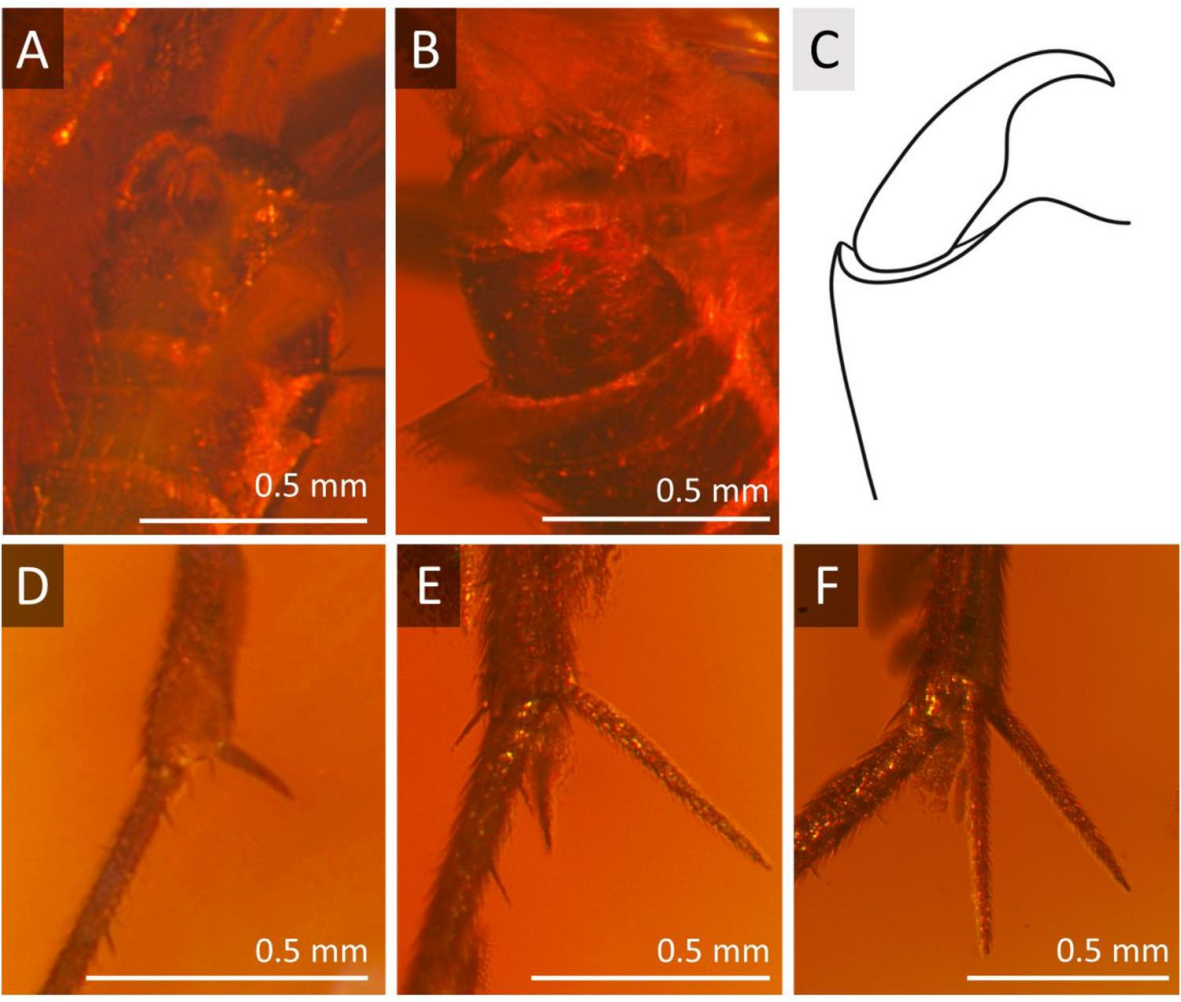
*Robsonomyia henningseni* sp. nov. (NHMD-39356): (**A**) male genitalia from ventral side; (**B**) genitalia from dorsal side, (**C**) drawing of genitalia from dorsal side; (**D**) apical spur on fore tibia; (**E**) apical spurs on mid tibia; (**F**) apical spurs on hind tibia.

***Robsonomyia henningseni*** Pełczyńska, Krzemiński et Blagoderov, **sp. nov.** (Figs. 4, 5, 6).

urn:lsid:zoobank.org:act:7CBF1E33-4650-44FC-83E6-7A143763E20D.

**Etymology:** The species name is derived from C.V. Henningsen, who donated over 3,000 amber pieces with inclusions to the Natural History Museum of Denmark. The holotype of this species was collected by him on January 16^th^ 1961.

**Type material:** Baltic amber inclusion No NHMD-39356 (Fig. 4A); preserved in 10 × 8 × 5 mm piece of amber (Fig. 4B).

**Diagnosis:** vein R_1_ terminates distinctly after M_1+2_ forking into M_1_ and M_2_; vein R_2+3+4+5_ strongly arched from half of its length towards anterior of the wing; M_1_ 3.1 × longer than M_1+2_; between Rs and m-cu the basal part of M_3+4_ is present, in the form of transverse basale (tb vein); gonostylus cylindrical, wide at the base, strongly narrowed and gradually arching inwards in apical half, pointed at apex.

**Description:** body 2.6 mm long, wing length 1.8 mm, antennae 1.3 mm long; Head (Fig. 5A): subspherical, wider than long; eyes large, well separated; membranous area separates ocellar sclerite from frons; large distinct cerebral sclerite present; three ocelli forming equilateral triangle. Antennae (Fig. 5B): about 0.7 × of wing length, about 0.5 × as long as body; scapus slightly shorter than broad, 1.4 × wider than flagellomeres; pedicel with bulbous apical part, as broad as scape; flagellum 14 segmented; flagellomeres cylindrical, densely covered with short setae, elongated, approx. 1.2 × longer than broad; terminal flagellomere slightly elongate, evenly tapered to rounded apex, 1.5 × longer than proximal flagellomeres. Palpi with three visible maxillary palpomeres. Wings (Fig. 5D, E): 2.4 × longer than wide, membrane hyaline without macrotrichia and without any visible markings; costa ending close after M_1_ vein reaches wing margin; Sc short, reaching Rb approximately at level of m-cu reaching Cu; R_1_ ending just after mid-length of anterior margin of the wing, distinctly after level of M_1+2_ forking into M_1_ and M_2_; R_2+3+4+5_ strongly arched anteriorly, second half of the vein runs almost in parallel with C; cross-vein m-cu 0.9 × as long as the radio-medal fusion (frm), between Rs and m-cu basal part of M_3+4_ is present (transverse basale = tb); Rs distinct, oblique, ending on the level of tb; Mb absent; M_1+2_ 2,3 × longer than frm, ending at the level of half the distance between where A and Cu terminates; Cu and A_1_ reaching wing margin; A_2_ absent. Thorax (Fig. 5C): about as high as long, scutum densely covered with long and thick hairs; anepisternum and katepisternum bare; mediopleural suture almost straight and subvertical; mesepimeron and laterotergite bare, mediotergite round and bare; haltere longer than first abdominal segment. Legs (Fig. 6D-F): fore coxa sparsely covered with long hair-like setae, mid and hind coxa with only a few setae; femora densely covered with short, robust setae; fore tibia with single apical spur, anterior tibial comb absent, mid and hind tibia with two equal length spurs, more robust and longer on hind tibia. Abdomen: densely covered with long hairs, all eight segments visible, I segment short, segments II-IV approx. same length, following segments gradually decreasing in length, segment VIII retracted into VII; male terminalia (Fig. 6A-C): tergite IX almost as long as broad, subcylindrical, slightly narrower at apex; gonocoxites massive, fused ventrally, almost straight at the apical margin ventrally; gonostylus cylindrical, wide at the base, strongly narrowed in its second half, curving gradually, pointed at apex; aedeagus and the associated internal structures not visible.

## Discussion

The decision to include the newly discovered species in Robsonomyiini was primarily based on the structure of the head. A unique apomorphy present in both species were observed, a membranous area separating the ocellar sclerite and the frons (Fig. 2A). In both species, the space between the isolated sclerites is narrow, but this may be a consequence of preservation and deformation during the fossilisation process. In addition, the cerebral sclerite is large and posteriorly extended, but not strongly defined and divergent from the head, which is typical of the tribe. Other features common to Robsonomyiini were found in the thorax (mediopleural suture is non-sinusoidal and subvertical), legs (lack of anterior tibial comb) and male genitalia (gonocoxites are fused and almost straight at the apical margin ventrally, whereas in Macrocerini they are usually distinctly concave). While the placement in the genus *Robsonomyia* itself was determined by the wing venation with a very characteristic shape of the Sc vein ending on Rb instead of terminating on the costa (diagnostic feature of the genus)^10,14^.

**Figure 7:**
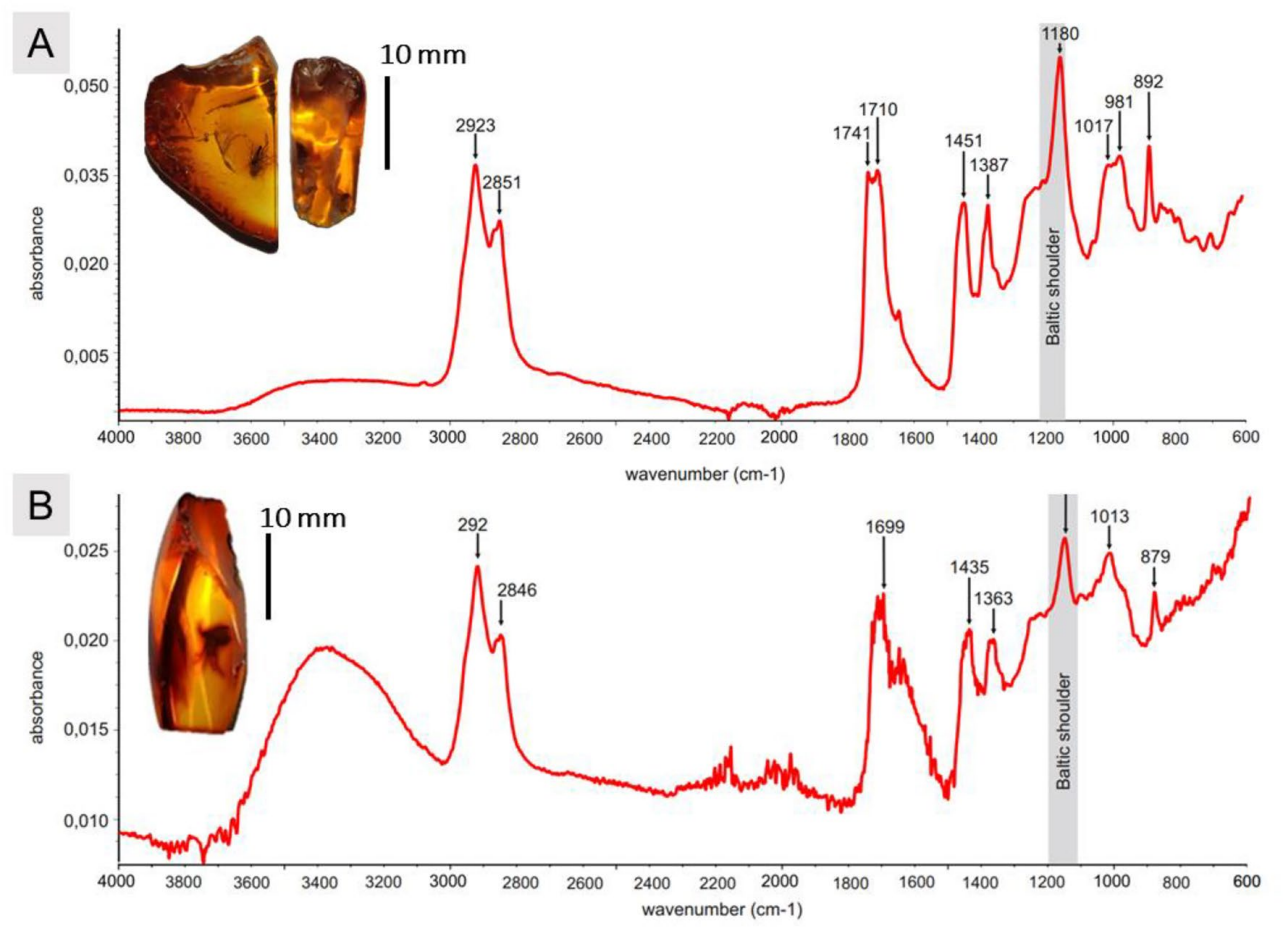
ATR-FTIR spectra of amber specimens with ’Baltic shoulder’ marked: (**A**) NHMD-300551 (**B**) NHMD-39356.

The fossil *Robsonomyia* species described here significantly improve our knowledge of the biogeographic history of the genus, expanding its current distribution. The Baltic origin of the specimens was confirmed with transform infrared spectroscopy analysis. The FTIR spectra showed distinctive features characteristic of the Baltic amber, including the ”Baltic shoulder” observed in the range 1190–1280 cm^−1^, accompanied by a strong absorption peak at 1170 cm^−1^ (Fig. 7)^18^.

**Figure 8:**
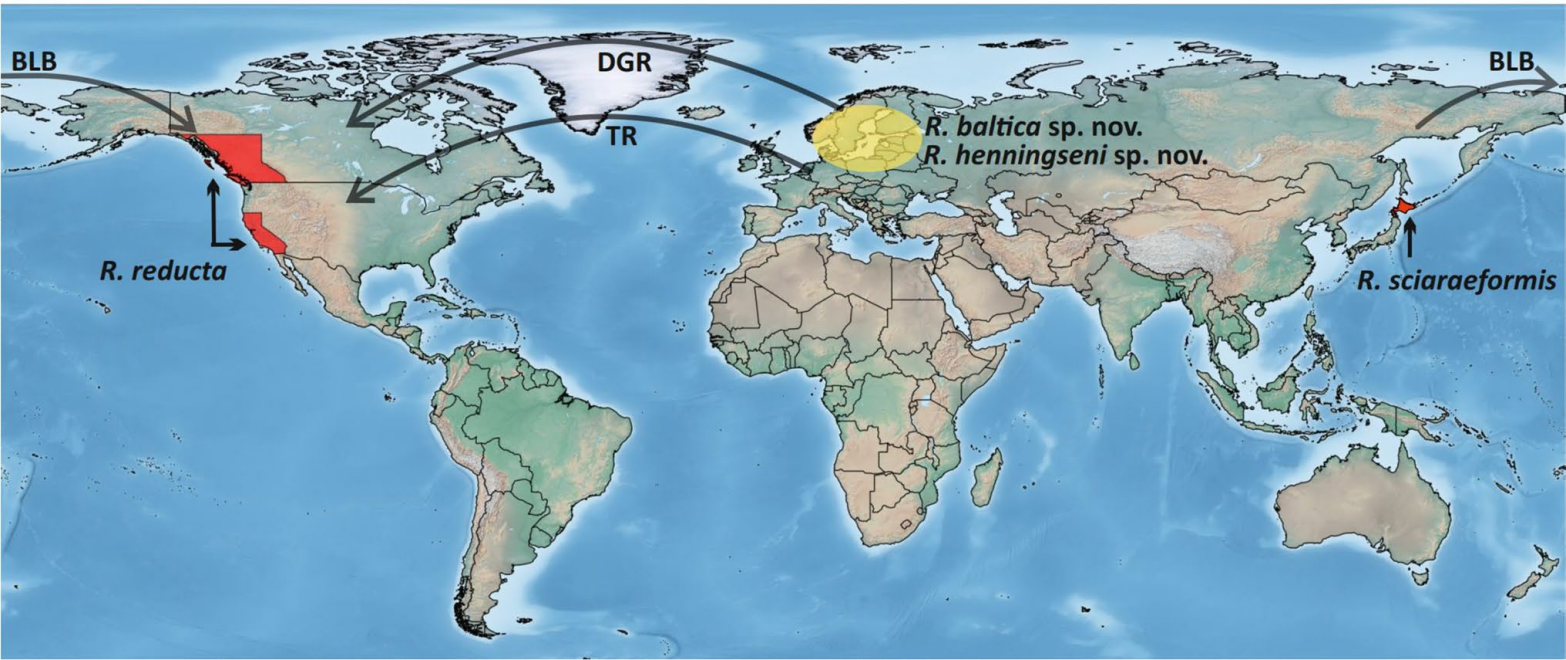
Geographical distribution of recent and fossil species of *Robsonomyia* (red—recent, yellow—extinct) with possible pathways of their dispersion marked: BLB—Bering Land Bridge, DGR—De Geer route, TR—Thulean route. Map created with SimpleMappr online generator (simplemappr.net) and modified with CorelDRAW 2018 (coreldraw.com/en/product/coreldraw).

This proved presence of *Robsonomyia* in Europe during the Eocene allows us to hypothesize that the current disjunct distribution is the relict of an earlier wider Holarctic distribution (Fig. 8). This pattern of occurrence is reminiscent of numerous other groups that were once widespread in the northern middle latitudes during the initial stages of the Tertiary period. For example, the disjunct pattern of distribution between Eastern Asia and Eastern Palaearctic and Nearctic exists in at least 65 genera of flowering plants that went extinct in the western Eurasia most likely due to orogenic events and climate change at the end of the Tertiary and during the Quaternary^19^. The classic example of this distribution pattern is represented by ginseng (Araliaceae: *Panax*)^20^. Among insects, the scorpionfly family Panorpodidae (genus *Panorpodes*) represents a similar case to *Robsonomyia*. Extant panorpodids are currently found only in eastern Asia (Japan, Korea and China) and North America but four species have been discovered in Baltic amber^21^. In the family Keroplatidae, there are genera that are now exclusively Nearctic, but for which amber records indicate a past Holarctic distribution. This is the case of *Palaeoplatyura* Meunier, 1899 (Keroplatinae) and *Hesperodes* Coquillett, 1900 (Macrocerinae)^1^.

**Figure 9:**
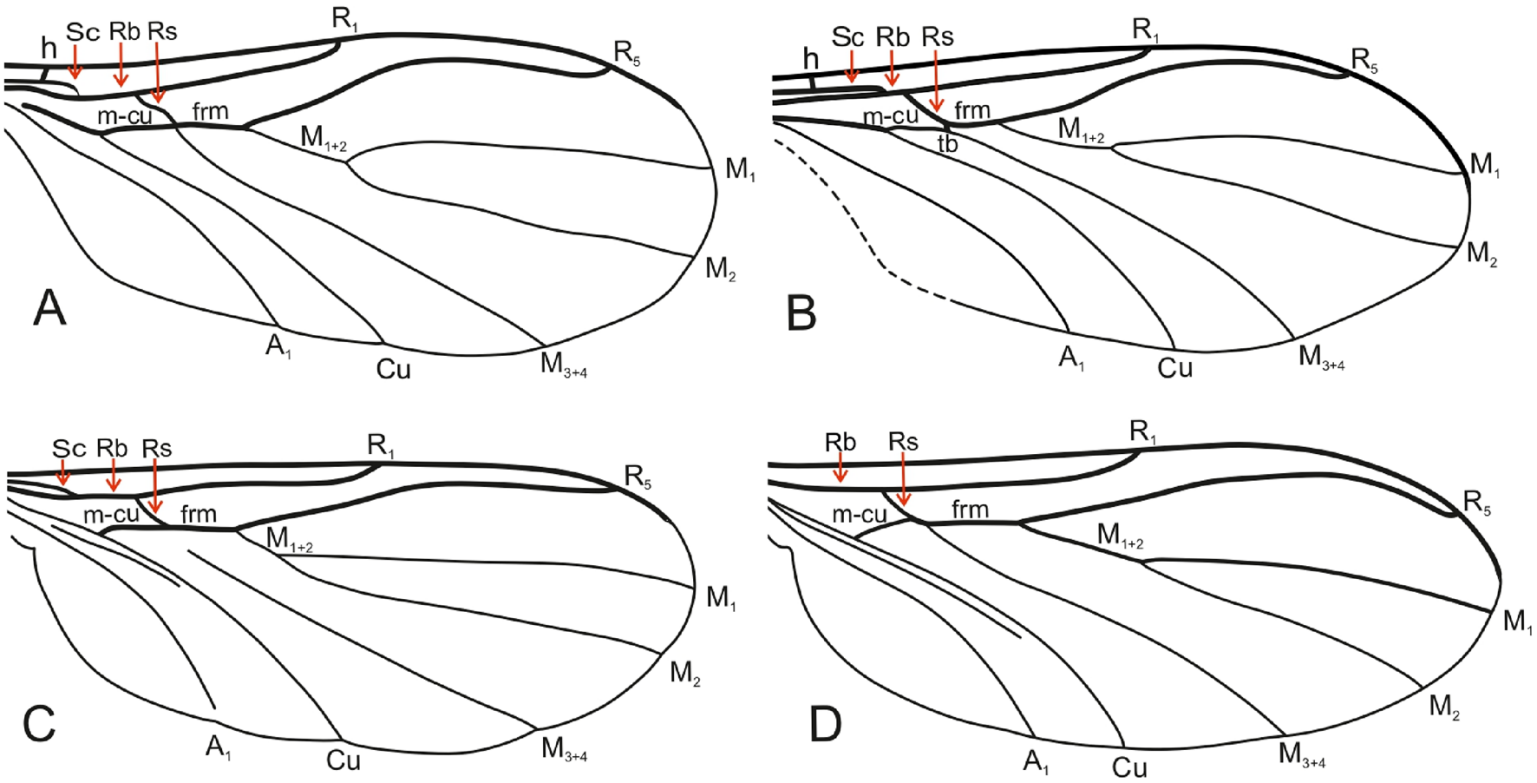
Wing venation of *Robsonomyia*: (**A**) *R. baltica* sp. nov.; (**B**) *R. henningseni* sp. nov.; (**C**) *R.* *reducta* (h vein not included in original drawing, after Matile & Vockeroth 1980); (**D**) *R. sciaraeformis* (Sc vein and h vein not included in original drawing, after Okada 1939).

**Figure 10:**
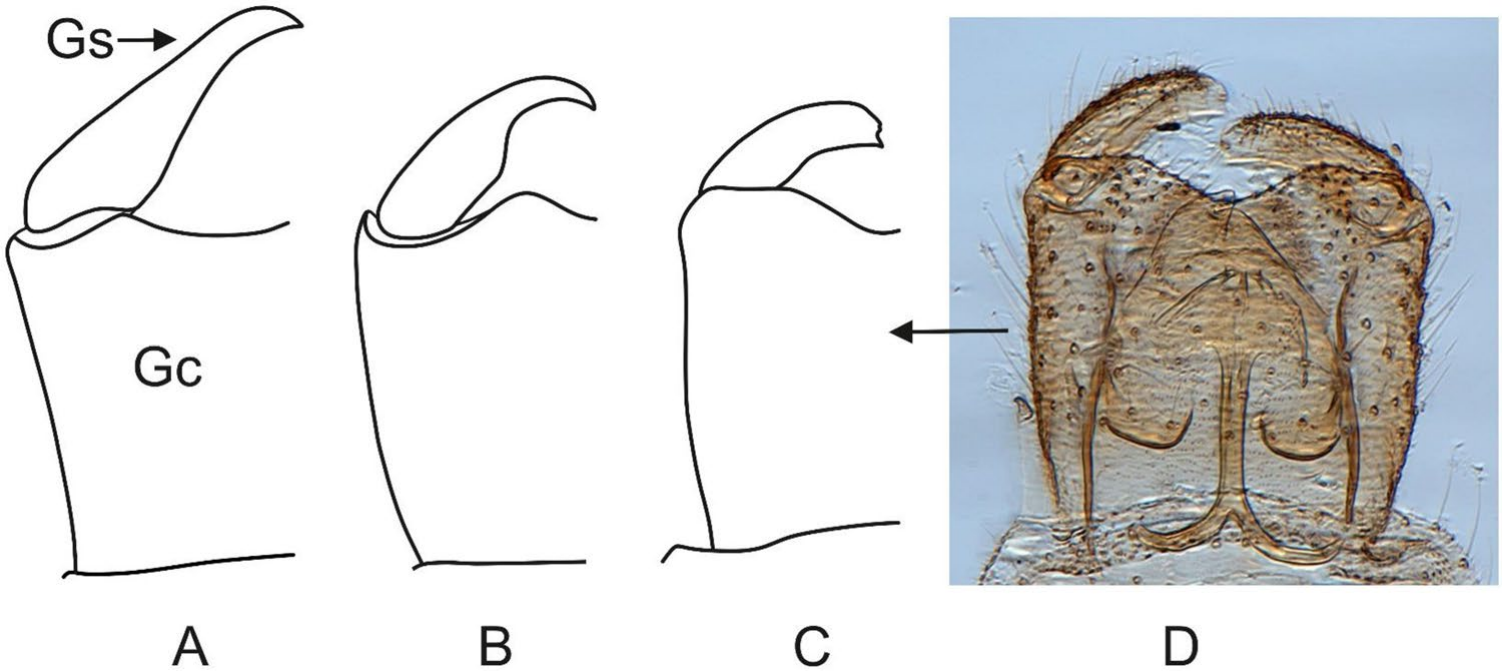
Male genitalia in *Robsonomyia*: (**A**) *R. baltica* sp. nov.; (**B**) *R. henningseni* sp. nov.; *R. reducta*: (**C**) drawing (**D**) photograph by Scott Brooks (Canadian National Collection of Insects, Ottawa). Genital structure in *R. sciaraeformis* not included due to lack of data.

By comparing representatives of *Robsonomyia* between each other we can observe morphological differentiation (Table 2) present in the wing venation (Fig. 9), the length of antennae and structure of genitalia (Fig. 10). A feature common to all species is the subcostal vein that joins the Rb vein although in *R. baltica* sp., it weakens considerably at the apex (Fig. 9A). Greater variation is observed in the medial sector. The presence of the tb crossvein (*transverse basale*) is observed only in *R. henningseni* sp. nov. (Fig. 9B, Table 2). In the other species, vein tb is absent, which is an apomorphic characteristic. Moreover, absence of the basal part of the M_3+4,_ separating vein from the m-cu cross vein is observed in *R. reducta* (Fig. 9C, Table 2). Notably, the anal sector of this species displays an additional apomorphy as anal vein (A_1_) does not reach the edge of the wing (Table 2).

**Table 2:** Selected morphological characters of *Robsonomyia* species.

Taxon	Characters							
	1	2	3	4	5	6	7	8
*R. baltica* sp. nov	0	1	1	0	0	1	0	1
*R. henningseni* sp. nov	1	0	1	1	0	1	1	1
*R. reducta* Matile & Vockeroth, 1980	?	1	0	0	1	0	1	0
*R. sciaraeformis* (Okada, 1939)	1	1	1	0	1	1	1	?

Character: (1) antennae reaching first abdominal segment, (2) R1 ending before M fork, (3) basal part of M3 + 4, (4) tb vein, (5) Cu ending before R1, (6) A1 reaching wing margin, (7) gonostylus shorter than gonocoxites, (8) gonostylus pointed at apex. Legend: 0—Character absent, 1—Character present, ?—Insufficient description provided by author.

Furthermore, there are notable differences in the structure of the male genitalia (Fig. 10, Table 2). The gonostylus can be long and exhibit a simple, cylindrical shape with a pointed apex (as seen in *R. henningseni* sp. nov. and *R. baltica* sp. nov.; Fig. 10A, B) or be short and flattened with a wide and blunt tip (as observed in *R. reducta*; Fig. 10C1, C2). The gonostyli of Sciaroidea, in their most basic layout, are simple, cylindrical tubes, that are closed at the apex^10,22^. Accordingly, any modifications such as shortening or thickening can be considered as apomorphic features^10,23^.

The finding of two species of genus Robsonomyia in the Eocene Baltic amber will certainly support future phylogenetic analyses, both in terms of dating the clades and in terms of enriching diagnostic features. The species found in Baltic amber suggest that *Robsonomyia* appeared at the latest in the Eocene. The current distribution of the genus is relictual and could be a vicariant pattern resulting from the subdivision of an ancestral wide distribution range followed by extinction in the western Palearctic. Alternatively, *Robsonomyia* could have dispersed out of the western Palearctic across Eurasia and further to east North America across one of the Beringian land bridges (BLB) that have intermittently connected these continents, or west across the North Atlantic, which was less extensive in the Eocene across, e.g., the Thulean route (TR) or earlier by De Geer route (DGR) (Fig. 8)^24,25^, or both. Additional information from fossils in North America and/or East Asia and a dated phylogeny for Macrocerinae is essential to test the probability of these different scenarios.

## Material and methods

The specimens examined were found in the Baltic amber, a fossil resin of Eocene origin with an age span from the Lutetian to the Priabonian (47.8‒33.9 Ma)^9^. However, the precise age of the amber remains unknown. The main reason is secondary redeposition; the amber has been transported and dispersed across the Northern European Plain, due to inter alia marine transgression and glaciers during the Pleistocene^26,27^. Consequently, the amber is not found in its original sedimentary context, and its stratigraphic history remains elusive^28^. Additionally, Baltic amber lacks radiogenic isotopes with long-term half-lives, preventing the direct application of radioisotopic dating methods for precise age determination^26^. The age of Baltic amber has been a subject of extensive debate, leading to the implementation of various methods that have yielded different results. For instance, glauconite dating has suggested a middle Eocene (Lutetian) origin for the amber, whereas microfossil dating has indicated a late Eocene (Priabon) timeframe^29^.

In this study, a total of four keroplatid inclusions were examined (2A, C, D and 5C). These inclusions consisted of one male and two females found within a single piece of amber NHMD-300551 (*Robsonymia baltica* sp. nov.), as well as one additional male from a separate piece NHMD-39356 (*Robsonomyia henningseni* sp. nov.). The specimens are housed in the collection of the Natural History Museum of Denmark (NHMD) in Copenhagen. Piece NHMD-300551 was cut in two during preparation for study, one piece containing the male holotype (NHMD-300551a) and the other containing two female paratypes (NHMD-300551b, NHMD-300551c). To enhance the visibility of the inclusions, the amber pieces underwent preparation, involving cutting, grinding, and polishing. To validate the authenticity of the fossil material, a Fourier transform infrared spectroscopy (FTIR) analysis was conducted. The analysis employed a Nicolet iS5 FTIR spectrometer, which was equipped with a diamond crystal attenuated total reflectance (ATR) attachment. The recorded spectra have been archived within the database of ISEA PAS as recommended for museum material by Zakrzewska et al*.* (2020)^16^.

Photographic documentation was performed using a Leica M205 C stereomicroscope equipped with a Leica DMC5400 camera. Focus stacks were acquired and processed in Leica Application Suite X (LAS X) (leica-microsystems.com/products/microscope-software/p/leica-las-x-ls). Drawings were generated by tracing photographs in CorelDRAW 2018 software (coreldraw.com/en/product/coreldraw). Additionally, a distribution map was created using the SimpleMappr online generator (simplemappr.net) and then modified using CorelDRAW 2018. The terminology used in this publication follows Matile (1990) with alternations in wing vein terminology after Sevcik et al. (2022)^10,17^. Modifications include: Rs = Rr; R_2+3+4+5_ = R5; M_3+4_ = M_4_; Cu1B = A_1_. Boundaries of zoological realms in Table 1 follow Evenhuis (2006)^1^.
